# Assessing the Impact and Cost-Effectiveness of Exposome Interventions on Alzheimer’s Disease: A Review of Agent-Based Modeling and Other Data Science Methods for Causal Inference

**DOI:** 10.3390/genes15111457

**Published:** 2024-11-12

**Authors:** Shelley H. Liu, Ellerie S. Weber, Katherine E. Manz, Katharine J. McCarthy, Yitong Chen, Peter J. Schüffler, Carolyn W. Zhu, Melissa Tracy

**Affiliations:** 1 Department of Population Health Science and Policy, Icahn School of Medicine at Mount Sinai, New York, NY 10029, USA; 2 Department of Environmental Health Sciences, University of Michigan, Ann Arbor, MI 48109, USA; katmanz@umich.edu; 3 Institute of Pathology, Technical University of Munich, 81675 Munich, Germany; 4 Munich Data Science Institute, 85748 Garching, Germany; 5 Department of Geriatrics, Icahn School of Medicine at Mount Sinai, New York, NY 10029, USA; 6 Department of Epidemiology and Biostatistics, State University of New York at Albany, Albany, NY 12222, USA; mtracy@albany.edu

**Keywords:** data science, agent-based modeling, exposome, Alzheimer’s disease, dementia, causal inference

## Abstract

**Background:** The exposome (e.g., totality of environmental exposures) and its role in Alzheimer’s Disease and Alzheimer’s Disease and Related Dementias (AD/ADRD) are increasingly critical areas of study. However, little is known about how interventions on the exposome, including personal behavioral modification or policy-level interventions, may impact AD/ADRD disease burden at the population level in real-world settings and the cost-effectiveness of interventions. **Methods:** We performed a critical review to discuss the challenges in modeling exposome interventions on population-level AD/ADRD burden and the potential of using agent-based modeling (ABM) and other advanced data science methods for causal inference to achieve this. **Results:** We describe how ABM can be used for empirical causal inference modeling and provide a virtual laboratory for simulating the impacts of personal and policy-level interventions. These hypothetical experiments can provide insight into the optimal timing, targeting, and duration of interventions, identifying optimal combinations of interventions, and can be augmented with economic analyses to evaluate the cost-effectiveness of interventions. We also discuss other data science methods, including structural equation modeling and Mendelian randomization. Lastly, we discuss challenges in modeling the complex exposome, including high dimensional and sparse data, the need to account for dynamic changes over time and over the life course, and the role of exposome burden scores developed using item response theory models and artificial intelligence to address these challenges. **Conclusions:** This critical review highlights opportunities and challenges in modeling exposome interventions on population-level AD/ADRD disease burden while considering the cost-effectiveness of different interventions, which can be used to aid data-driven policy decisions.

## 1. Introduction

Alzheimer’s Disease and Alzheimer’s Disease and Related Dementias (AD/ADRD) are estimated to affect over 6.7 million Americans, and remains the fifth leading cause of death among people aged 65 and older in the US [1]. Only 10–30% of AD/ADRD risk is attributed to genetics [1]. For example, mutations in several genes (e.g., amyloid precursor protein, presenilin 1, and presenilin 2) [2] are known causes of early onset AD, while some genes and gene variants such as Apolipoprotein E (*APOE*) are associated with an increasing risk for developing late-onset AD [3,4,5,6,7,8,9]. Meanwhile, the environment is estimated to contribute 70–90% to AD/ADRD disease risk [1]. The exposome, or the totality of environmental exposures an individual encounters throughout their lifetime from conception onward, is a paradigm that was created to describe lifetime exposure risk [2,10,11]. The exposome encompasses all non-genetic factors that contribute to health and disease, providing a more holistic view of the factors influencing human health than genetic data alone. The exposome includes a myriad of environmental influences, ranging from chemical agents in the air, water, and food to social, psychological, and lifestyle factors [3,4,5,6,7]. These exposures interact dynamically with the human body, influencing biological processes and contributing to disease risk [8,9,12,13,14]. 

The exposome that affects Alzheimer’s disease is diverse, including exogenous factors (e.g., environmental toxicants, light and noise, climate, ecosystems, and economics/education), endogenous factors (e.g., genes/epigenetics, pre-existing conditions, metabolism, microbiome, and xenobiotics), and behavioral factors (psychological effects, drugs/alcohol/tobacco, lifestyle, sleep, and stress) [13]. Many studies have linked AD/ADRD with structural/social determinants [14] such as food access [15,16], greenspace [17,18], recreation [19,20], transportation [21,22], housing [23,24,25], poverty [26,27], policing and incarceration [28,29], neighborhood deprivation [30,31], workplace/occupation [32,33], income [34,35], literacy [36,37], education [38,39], health care [40,41], social networks, isolation, and loneliness [42,43]. Environmental biotoxins such as spirochetes, toxic molds, periodontitis, viral infection [44], and environmental metal toxins [45,46,47,48] have also been found to be involved in AD development and progression. Other environmental pollutants [49], including industrial and commercial pollutants (e.g., dioxins [50,51,52], bisphenol [53,54], phthalates [55,56], brominated flame retardants [57,58,59], alkylphenol polyethoxylates [60,61]), air pollution (e.g., particulate matter [62], ozone [63], volatile organic compounds [64]), and pesticides [65,66,67,68,69,70] were found to be directly or indirectly involved in AD pathogenesis. An increasing number of studies [71] have found that light pollution influences AD [72,73,74,75,76,77,78].

Building on the associations that have been identified thus far between the chemical and non-chemical exposome and AD/ADRD, there is emerging interest in the influence of the cumulative exposome burden on the onset and progression of AD/ADRD. To date, it has been difficult to estimate the impact of the cumulative exposome in real-world settings. In this critical review, we will use a rigorous quantitative perspective to discuss the challenges inherent in modeling the exposome and its effects on AD/ADRD. We will discuss the potential of advanced data science techniques such as agent-based modeling (ABM) and other causal inference data science methods to simulate and measure the effects of intervening on the exposome. Furthermore, we will describe the need to incorporate cost-effectiveness evaluations of interventions aimed at reducing exposome burdens, thereby considering their economic impact alongside their potential to mitigate disease burden.

## 2. Challenges in Modeling the Exposome over the Life Course and Its Impacts on AD/ADRD

The onset and progression of AD/ADRD are complex and involve multiple susceptibility genes and environmental factors [79]. Modeling environmental factors is challenging because they evolve over time. Individuals can encounter different environmental factors and exposures at various life stages, each with a unique impact. Prenatal exposure to chemicals can have long-lasting developmental effects [34,35,36,37], while adulthood exposures may impact disease progression [38,39,40].

Gene–environment interactions are also critical for understanding the AD/ADRD mechanisms and developing personalized intervention strategies [80]. Precision environmental health aims to understand the complex interactions between the exposome and individual genetic susceptibility so that personalized interventions may be designed to prevent adverse health effects [17]. By integrating environmental exposures with large system-level (“omic”) datasets [81], precision environmental health can enhance our understanding of underlying environmental causes of AD/ADRD and develop personalized prevention and intervention strategies. Recognizing that an individual’s response to environmental exposures is highly variable, the integration of an individual’s personal “omics” data (i.e., metabolomics, proteomics, epigenomics, etc.) may improve the ability to identify specific disease risk factors so that interventions can be tailored to mitigate adverse health outcomes.

Modeling methods to date have traditionally drawn on epidemiological data sources to test targeted hypotheses about the association of independent exposures and outcomes [82]. The limitations of traditional approaches are that exposures frequently co-occur or are collinear, making it difficult to isolate an independent effect within a complex, dynamic network [83]. Exposures also often interact amongst themselves—for example, either environment-by-environment or gene-by-environment. Another current challenge in exposome-AD/ADRD research is the lack of data availability in which both the exposome and neurophenotyping data are well-characterized in the same study. The use of complex systems approaches, such as ABM, can be a valuable tool that, through the simulation of synthetic agents, can leverage comprehensive environmental data and longitudinal neurophenotyping data from different data sources to capture the exposome and examine its relationship with health measures.

## 3. Agent-Based Modeling for Modeling the Impact of Exposome Interventions on Population-Level AD/ADRD Burden

Agent-based modeling (ABM) is a computational modeling approach that generates population-level phenomena from simple rules governing individual behaviors (“agents”) and interactions [84,85]. It is a useful tool for exploring the macro-level implications using micro-level assumptions [86]. Agent-based approaches originated in ecology, social science, and anthropology studies [87,88,89] and have been used in bioinformatic studies for analyzing the potential interactions of biological elements, understanding complex biological processes, and predicting behaviors under various conditions [90]. In public health, ABM was initially used to model the spread of infectious diseases in a population [91,92], and is now increasingly used to investigate other health-related conditions, including chronic diseases [93] and violence [94], as well as the context that gives rise to health behaviors and outcomes, including the food retail environment [95] and socioeconomic inequalities [96]. The defining feature of ABMs is their incorporation of individual heterogeneity, meaning that each individual “agent” in the model has their own diverse characteristics that influence their behaviors and experiences, which in turn produce population-level behaviors and outcomes [97,98]. The individual heterogeneity captured in ABM is a major strength in the era of personalized medicine and stands in contrast with other complex systems modeling approaches, like system dynamics (SD) modeling, which focuses on aggregate dynamics [99]. In addition to shedding light on the mechanisms that give rise to population-level patterns, ABMs can be used as virtual laboratories, simulating potential interventions at both the individual and population levels [100]. These hypothetical experiments can provide insight into the optimal timing, targeting, and duration of intervention conditions, as well as the optimal combination of interventions and policies, to address the population health problem being studied. These models can also incorporate potential costs [101] and anticipate unexpected consequences [102], thereby serving as the basis for recommendations regarding resource allocation and the implementation of policies, regulations [103], and interventions.

Several recent studies have explored the potential of ABM for modeling the exposome. These studies, aimed at creating a framework to model the totality of human exposures based on daily activity patterns, take advantage of many of the strengths of ABM, including explicitly modeling individual heterogeneity in a synthetic population and embedding individuals in a virtual physical environment that can vary from a simple abstract grid to a hyper-realistic representation of specific locations, using geographic information systems (GIS). Two such examples, the SpatioTemporal Human Activity Model (STHAM) and the Agent-Based Model of Human Activity Patterns (ABMHAP), have generated realistic longitudinal human activity patterns for different demographic groups (e.g., working adults, non-working adults, school-age children, and preschool-aged children) [104,105,106]. These patterns have been validated against daily activity survey data [105] and traffic patterns [104]. Although these models can be extended to map activities and locations to specific indoor and outdoor exposures, current versions include a limited range of activities and do not consider interactions between people when generating activity patterns [104,105]. Another ABM by Chapizanis and colleagues combined data from a variety of sources, including population, time-use, road network, and air quality data, to create a model of urban Thessaloniki, Greece, including longitudinal trajectories of human behavior that were validated against data from wearable sensors [107]. This model identified population sub-groups with the highest exposure to PM_2.5_ concentrations and highlighted variability in exposure levels between people, even those living near each other. Similarly, Novak and colleagues developed an illustrative ABM to reproduce patterns of PM_2.5_ exposure based on data collected through personal monitors, using an abstract representation of the environment and simple activity rules [108]. These and other recent models extend traditional applications of ABM investigating pathogen exposure in healthcare facilities [109] and during hypothetical bioterrorist events [110] to consider broader environmental exposures, including exposure to fine particulate matter [111] and contaminants in the water distribution system [112]. Together, this work has provided insights into prevention and management strategies for exposure threats, as well as the important role of individual decisions and behaviors.

Currently, however, few ABMs have evaluated the human health effects of the exposome or the role of environmental exposures on the onset and progression of AD/ADRD. An ABM of traffic-related air pollutants developed by Hyesop Shin connected a fairly detailed representation of commuting patterns in Seoul, South Korea, to a fairly abstract measure of health risks associated with non-exhaust PM_10_ emissions, operationalized as a nominal health index with a cut-off to identify individuals “at-risk” of poor health [113]. Separately, some recent ABMs have been used to examine the development of AD/ADRD, highlighting the role of microbial initiation of late-onset Alzheimer’s disease via the olfactory system [114], cellular pathways that contribute to neurodegeneration [115], and the potential for blood pressure-management strategies to prevent or delay AD/ADRD development [116]. These findings hint at the potential for ABM to uncover key exposures and processes related to the development of AD/ADRD. However, no ABM studies to date have connected the exposome with AD/ADRD onset or progression.

In order to take full advantage of ABM approaches for models of the exposome and its effects on AD/ADRD burden in the population, these models need to capture dynamic exposures and substitutions in exposures that may occur over the life course, including because of interactions with other people and in response to changing regulations and industrial practices. ABM also explicitly models adaptations in behavior in response to environmental changes, social norms and peer influence, and past experiences [117]. Although sensitivity analyses can be used to test different assumptions about unknown parameters and dynamics, some reliable empiric data about the longitudinal processes under study are helpful to calibrate and validate these models, especially when promoting their use for policy recommendations [100,117]. ABM can incorporate data from a variety of different sources to represent the underlying system in question, which can be particularly useful when well-characterized exposome and neurophenotyping data are not available in the same study. Further, ABMs are highly flexible, enabling representations of non-linear dynamics, non-additive relationships, latent constructs of cumulative exposure, and interactions across multiple levels of influence, including gene–environment interactions. However, models can quickly become quite complex, necessitating careful deliberation about the critical aspects of the system that must be included [102,118].

As noted above, a major strength of applying ABM to the exposome and AD/ADRD is the opportunity to evaluate potential intervention and treatment effects, including comparing strategies targeted to the individual vs. policy level, as well as different combinations of interventions. ABM studies of hypothetical policy scenarios have shed light on industry responses to water-management policies [119]; the dynamics of air emissions cap and trade programs [120]; and the relative impacts of different policies, including education and economic incentives, on urban residents’ PM_2.5_-reduction behavior [121]. ABM studies have also been used to inform optimal resource allocation, including in the event of bioterrorism threats [110,122] and contamination of the water supply [112]. These models have also demonstrated how much intervention effects may vary according to the environmental context and individual characteristics [119,123,124], highlighting the importance of capturing this individual and environmental heterogeneity to make informed predictions.

**Figure 1:**
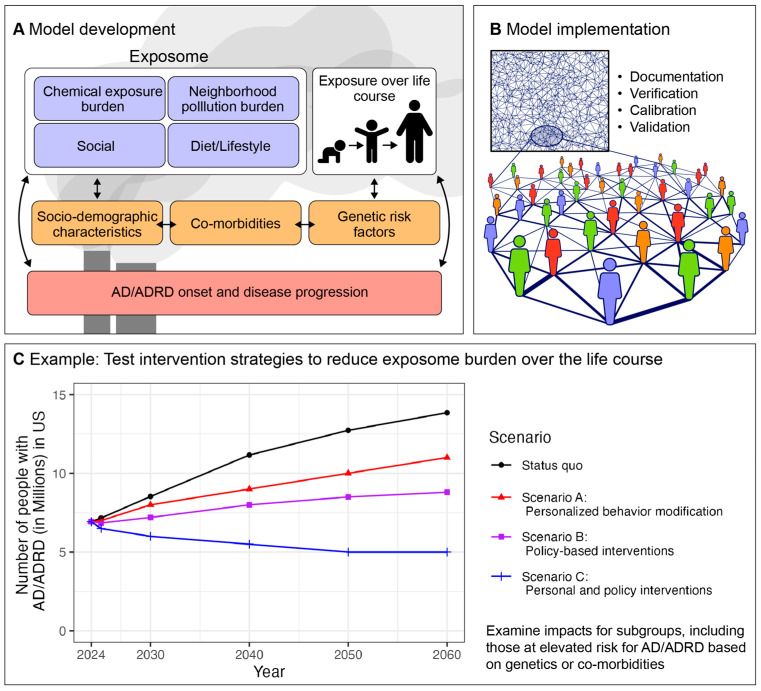
A conceptual diagram of agent-based models used to determine exposome impacts on population-level AD/ADRD burden.

Figure 1 illustrates how we can use these models to simulate the impacts of different interventions on population-level AD/ADRD burden. A population of synthetic agents is developed using known information about the exposome, including chemical exposure burden, neighborhood pollution burden, social and dietary/lifestyle behaviors, as well as socio-demographic characteristics, co-morbidities, and genetic risk factors from the literature or from existing data. For example, the synthetic agents can represent the overall US population, characterized using nationally representative exposome biomonitoring and census data. After calibrating and validating the dynamics of exposome changes and health outcomes in this synthetic population, we can then test interventions to identify the impact on the population-level AD/ADRD burden in the US. The status quo scenario is based on projections by Rajan et al. [125], with nearly 7 million people living with AD/ADRD in the US in the present day, which is projected to be 13 million by 2050. We can then test interventions across our population of synthetic agents to determine if they would result in reductions in the national AD/ADRD burden. These interventions may take place on the individual level (e.g., personal behavior modifications) or at the population level (e.g., policy changes). An example of an individual-level intervention may be to make dietary modifications or behavioral changes that reduce a person’s systematic exposure to plasticizers and other synthetic organic pollutants, for example, through increased home cooking and the use of personal filtration systems for drinking water. An example of a population-level intervention may be to set federal standards for drinking water contamination in municipal water systems, such as the standards currently proposed for per- and polyfluoroalkyl substances (PFAS). The cost-effectiveness of these potential interventions could also be considered by incorporating the cost of interventions (e.g., installing filtration systems) vs. their economic benefits on the population level.

Although ABM holds promise for providing insight into the potential effects of exposome interventions on population-level AD/ADRD, several challenges exist. First, harmonizing existing data, including data on environmental exposures, individual behaviors and activities, and intervention effects, across multiple time scales as inputs to an ABM is a major challenge [119]. Second, the best representation of the full range of interacting exposures that humans face throughout their lifetime, accounting for gene–environment interactions, remains unknown [126]. Third, the scalability of successful ABMs of the exposome to other locations will depend on the availability of similarly granular data on population activities and environmental exposures, as the exposome can be expected to vary greatly across locations and times [127]. Efforts to overcome these current limitations include novel approaches to measuring cumulative exposures that can inform ABM implementation [128], as well as the development of a model architecture that can represent different aspects of human–environment interaction [117]. Ongoing large-scale projects aimed at quantifying the exposome and its health effects, including the European Union-based HEALS project [129] and EXposome Powered tools for healthy living in urbAN Settings (EXPANSE) [130], may fill some data gaps and will likely be important resources for future ABM work. These approaches will complement other causal modeling approaches like structural equation modeling and g-computation in developing a more robust body of evidence about the effects of the exposome on health over the life course.

## 4. Other Causal Inference Methods to Quantitatively Model the Impact of Exposome Interventions

Several causal approaches may have particular relevance to modeling the exposome and gene–environment interactions. These include Mendelian randomization (MR) design, which exploits genetic variants as instrumental variables to estimate the causal effects of a wide array of risk factors on outcomes and can maximize big data sources [131,132]. Causal mediation analyses can shed insight into causal pathways through effect decomposition into direct and indirect effects and can accommodate exposure–mediator interaction [132]. Issues such as time-varying confounders that are affected by prior levels of exposure can also be addressed by using inverse probability weighting [133]. A recent study has applied the MR approach in evaluating the impacts of environmental exposomes on neurodegeneration and reported a higher risk of AD in people with lower educational attainment, higher weekly beer and cider intake, lower strenuous sports or other exercises, higher cigarette consumption per day, lower diastolic blood pressure, and lower body fat percentage [134].

Another approach is to use a structural equation model (SEM) framework to model complex systems and allow for reciprocal causation. This method estimates structural relationships between latent variables, which are inferred from one or more observed (measured) variables. SEM models allow investigators to test whether an underlying theoretical model is supported by model fit and the magnitude, direction, and statistical significance of specified pathways [135]. SEM has been applied in psychiatric research to study multifactorial models of cognitive disorders in complex systems, including early life exposures, behavioral factors, and neuro-imaging markers [136]. A limitation of SEM is that while this is a multivariable approach, data reduction may be warranted in the event of high amounts of exposome information. Approaches such as factor analysis, principal components analysis, or item–response theory can be used to reduce dimensionality and to map observed indicator variables onto latent exposome variables [137]. Several studies [138,139] have applied SEM in assessing the relationships between air pollution exposure and cognitive decline and reported associations between higher PM2.5 and increased AD risk [138], higher PM2.5 and poorer memory [139], and higher NO2 and poorer memory [139].

SEM approaches are primarily designed to model linear relationships. Newer advances in SEM, however, can accommodate non-linear relationships using latent growth curve modeling (LGCM). LGCM is a specialized version of SEM, which summarizes trajectories of exposome indicators into latent variables (i.e., “growth factors”). LGCM models use longitudinal data with indicator variables measured over time to estimate two types of latent variables—the intercept, or initial status of exposure, and its slope or rate of change over time. Pathways from the intercept to the intercept and slope at a subsequent time point can capture stability or change in growth parameters, providing insight into how variables evolve and interact within complex systems [140]. To leverage the correlation between growth factors, g-computation methods can be used to estimate the overall effect of the mixture of growth factors on neurocognitive outcomes [141]. Using these approaches, the significance of growth trajectories and their magnitude and direction of influence can contribute to understanding the underlying features of the exposome that are driving change.

## 5. Considerations of Economics and Cost-Effectiveness in Studying the Impact of Exposome Interventions

Economic evaluations help stakeholders and decision makers such as policymakers, healthcare providers, and payers to make informed choices about how to allocate limited resources to achieve the best possible health outcomes. The overarching purpose of an economic evaluation is to provide a structured analysis of the costs and outcomes of specific diseases and conditions. Most often, these are comparative analyses (such as cost-effectiveness analyses [CEA] and cost–benefit analyses [CBA]) that measure disease costs and outcomes under different healthcare interventions, but analyses can also be absolute and stand-alone (such as intervention cost, return on investment, and economic burden and budget impact analyses) [142].

Despite the growing economic literature on AD/ADRD, there have been few economic evaluations on the exposome. Very recently, Li et al. quantified the economic impact of ozone pollution on AD in China [143]. They found that ozone pollution contributed to almost 110,000 more new cases of AD in China in 2023 compared to 2015, imposing an economic cost of about US USD 1.2 billion. They also offer a threshold for ozone concentrations (70 μg/m [11]) that could prevent 210,000 new AD cases, potentially saving USD 2.2 billion. Of note, they do not use any economic modeling in their analysis, instead relying on a systematic review of the literature to determine the concentration–response coefficient and thus the change in AD and mild cognitive impairment costs from a decreased ozone concentration. In a similar analysis, Yang et al. [144] looked at daily exposure to airborne particulate matter in China and its impact on hospitalizations for AD, including the economic costs of such hospitalizations. Economic burden analyses such as these, which measure the financial impact of exposomic elements on AD/ADRD patients and thus to society, are valuable because they communicate the potential economic value of intervening in these factors. Thus, they can help inform decision making and resource allocation.

However, a more nuanced approach to informing the viability of exposome interventions would be to use a comparative approach, whereby multiple options are compared to determine which provides the best value for the money. This can include comparing new treatments to usual care or assessing different strategies for disease diagnosis [145] or management. Usually, CEAs use a standardized metric for effectiveness—a quality-adjusted life year or QALY—to facilitate comparison across interventions (for example, see Ross et al.’s study [146] on the cost-effectiveness of two anti-amyloid monoclonal antibodies in slowing the progression of AD). The use of QALYs enables comparison not only with alternative AD/ADRD treatments but also with interventions for other diseases or societal ailments. Findings can inform whether the health sector and society would do better allocating resources to other places [147].

The use of models is already fundamental in economic evaluations such as CEAs for simulating the trajectory of diseases (including AD/ADRD) over time and helping to predict costs and outcomes under different scenarios, especially where longer-term data are lacking [148]. These models may incorporate disease states, patient demographics, treatment efficacy, and healthcare resource utilization. Several types of models can be used in economic evaluations, including decision trees [149], survival partition models [150], Markov models [151], discrete event simulations (DES), microsimulation models [152], and ABMs [153]. These models vary in complexity and are chosen based on the nature of the disease, the intervention being evaluated, and the available data. One study [154] of the literature on pharmacological treatments for AD/ADRD found that Markov models, DES, and microsimulation were the most common. They concluded that insofar as all models were limited by their abilities to conceptualize and reproduce the course of AD, the key element was translating changes in cognition, function, and behavior into meaningful outcomes (e.g., QALYs, time to institutionalization, full-time care, and costs) for decision makers.

Currently, to our knowledge, there are no analyses incorporating ABMs into economic evaluations of AD/ADRD interventions. This is likely because ABMs, which overcome the limitations of other economic evaluation models [153], have not yet been needed. The literature so far has focused only on simple interventions and treatments, such as those involving single drugs or non-pharmaceutical protocols of limited duration. However, ABMs will be needed to evaluate potential exposome interventions and their associated effects, including cost impacts. ABMs can accommodate greater dynamism, higher dimensions, and greater individual variability than models of simpler disease interventions and their effects. This is necessary given the exposome’s multifaceted nature, the complex gene–environment interactions at play for AD/ADRD, and the longer time scales of exposome effects throughout the life course.

The estimation of costs and QALYs have previously been integrated into other micro-simulation models of dementia [155]. Representative population-level data from national health surveys (e.g., the Health and Retirement Survey, Medicaid Expenditure Panel Survey) or from claims data (e.g., Centers for Medicare and Medicaid Services data, MedStat, and MarketScan data) can help integrate costs and QALYs into an ABM of AD/ADRD. Developing an ABM that outputs costs and QALYs would enable apples-to-apples comparisons to other health or even non-health interventions. Decision makers need a uniform comparison metric to be able to know how to best allocate resources across competing interventions or policies.

## 6. Future Directions and Big Data Considerations of Modeling the Exposome and Impacts of Interventions on Disease Burden: Innovative Use of Exposome Burden Scores and Artificial Intelligence

As the exposome is complex and changing over time, future work in modeling interventions necessitates a harmonized metric of quantifying cumulative exposome burden. Precision environmental health has proposed the use of “multi-omic burden scores” of exposure burden for primary prevention of disease so that the interventions can be tailored to subgroups of the population with the highest exposure burden and/or the greatest vulnerability for the disease. However, quantifying the exposome burden into a single metric or set of summary metrics is challenging for multiple reasons: (1) the data are highly dimensional and often sparse, meaning that data on various facets of the exposome may not be available for all people due to the high cost of comprehensive data collection; (2) different subpopulations may have different dietary habits and behaviors that may systematically expose them more to different facets of the exposome; and (3) over time, the exposome may change due to both a person’s changing habits over their life course, as well as changes in the external environment (e.g., due to regrettable substitution, with different chemicals being phased out of production by industry and replaced by others, which are just as harmful to health). For example, we hypothesize that intervening on a specific aspect of the exposome, e.g., by using “BPA-free” water bottles, may reduce someone’s exposure to a specific plasticizer but not have a measurable impact on their overall exposure burden to plasticizers due to regrettable substitution. Regrettable substitution, which is the replacement of a toxic chemical with one that is later proven to be equally or more harmful, highlights a significant challenge in managing the exposome [31]. As another example, a proposed policy intervention on a specific air pollution component may not have the desired impact on the AD/ADRD disease burden if it is replaced or the levels of another harmful constituent increase.

Future work in this direction necessitates harmonized summary metrics that are interpretable across studies. Recognizing these challenges, Liu et al. [156,157] proposed novel applications of item response theory (IRT) to quantify exposome burden scores. Liu et al. demonstrated how IRT can be used to create a common exposure burden scale across studies, such that the summary metrics retain the same meaning and can be used for cross-study harmonization [128,156,157,158,159,160]. This is often necessary for studying the exposome because different studies may measure different aspects of the exposome, and the authors demonstrated that IRT can be used to make full use of all data, even if studies do not measure the exact same set of features, by using the common features as “anchors” to set a common scale. Meanwhile, using a proxy (e.g., monitoring a single chemical contaminant to represent cumulative impacts of an entire chemical class) may not be as informative because they do not account for the changing landscape of population-level exposure to chemical contaminants due to the phasing out and introduction of other chemical contaminants by the industry. With summary metrics, combinations of interventions, or interventions across an entire chemical class, can be tested, and impacts on subgroups, for example, those at greater genetic risk, can be modeled for studying gene–environment interactions at a population level.

New methods of artificial intelligence (AI) pose further innovative directions for the study of the exposome and their interventions. AI can detect relevant patterns in data that reflect a certain condition. Many of the above-mentioned studies employ stochastic models, rule-based models (simulations), and classical machine learning techniques such as supervised learning (e.g., regression) or unsupervised learning (e.g., clustering). In these scenarios, the confounding factors (i.e., the exposome) are directly related to a particular outcome (e.g., AD/ADRD), and the model learns the relationship between the input and the outcome. This approach requires historical experience via training data to learn the relationship. However, a new and largely unexplored field for exposome research is self-supervised learning (SSL) [161]. In SSL, deep neural networks learn intrinsic structures of the given data by differentiating between similar and non-similar data (contrastive learning) or by reconstructing data from noisy versions of themselves (non-contrastive learning). In both ways, the goal is not to solve a particular task, such as risk prediction, but to enable the model to capture and generate relevant information. For example, if a model can complete masked parts of a medical image, we assume the model has learned about the medical domain or the underlying disease. SSL has been used in the medical domain in a wide range of applications [162], mainly in tasks involving medical images [163], electronic health records [164], RNA/DNA Network data [165,166,167], or time series [168]. It is only natural that SSL will also lead to innovative studies in exposome research. SSL requires large-scale data sets to be trained, which poses the largest challenge in medical applications. But we are confident that soon, this will be solved, e.g., by merging the increasing number of available data sets and studies, thus reaching hundreds of thousands of participants. Like other generative models (ChatGPT, Gemini, Llama, or others), these generative exposome models could be used to predict probable hotspots for high-risk AD/ADRD disease or to explain the contribution of individual confounder factors to a given AD/ADRD prevalence with attention-based techniques.

In this critical review, we highlighted opportunities and challenges in modeling exposome interventions on population-level AD/ADRD disease burden while considering the cost-effectiveness of different interventions. There is a dearth of research in this field; thus, we showed how we can use novel combinations of approaches across data science, causal inference, and artificial intelligence to develop these research goals. We focused on describing how a unique combination of tools integrating empirical causal inference modeling using ABMs with economic analyses and contemporary machine learning methods for studying exposome–outcome associations, including exposome burden scores, can allow us to holistically simulate the impacts of exposome interventions. We believe these tools will be important for informing data-driven policy decisions and encourage interdisciplinary researchers to work together to advance this field. 

## Data Availability

No new data were created or analyzed in this study. Data sharing is not applicable to this article.
